# Utility of various positron emission tomography tracers in differentiating primary central nervous system lymphoma from glioblastoma multiforme

**DOI:** 10.1186/s41824-026-00302-x

**Published:** 2026-05-01

**Authors:** Nobuhiko Kawai, Nobuyuki Miyatake, Kenta Suzuki, Daisuke Ogawa, Tetsuhiro Hatakeyama, Yoshihiro Nishiyama, Keisuke Miyake

**Affiliations:** 1 Department of Neurological Surgery, Faculty of Medicine, Kagawa University, 1750-1 Ikenobe, Miki-cho, Kita-gun, Kagawa, 1750-1 Japan; 2 Department of Hygiene, Faculty of Medicine, Kagawa University, 1750-1 Ikenobe, Miki-cho, Kita-gun, Kagawa, 1750-1 Japan; 3 Department of Radiology, Faculty of Medicine, Kagawa University, 1750-1 Ikenobe, Miki-cho, Kita-gun, Kagawa, 1750-1 Japan

**Keywords:** Lymphoma, Glioma, Isocitrate dehydrogenase mutation, Hypoxia, [^18^F]fluoro-2-deoxy-D-glucose, L[methyl[^11^C]]methionine, 3′deoxy3′[^18^F]fluorothymidine, [^18^F]fluoromisonidazole

## Abstract

**Background:**

Primary central nervous system lymphoma (PCNSL) can be differentiated from glioblastoma multiforme (GBM) using positron emission tomography (PET) with [^18^F]fluoro-2-deoxy-D-glucose (FDG). However, differentiation is often difficult with magnetic resonance imaging (MRI) or FDG PET alone. We have used various PET tracers to aid glioma diagnosis; here, we assessed whether multiple PET tracers improve the distinction between GBM and PCNSL.

**Methods:**

We studied 148 patients with newly diagnosed brain tumors: 96 with GBM and 52 with PCNSL (according to the 2016 World Health Organization classification). Tumor-to-normal tissue ratios (TNRs) were calculated for FDG, L[methyl[^11^C]]methionine, and 3′deoxy3′[^18^F]fluorothymidine (FLT). Tumor-to-blood ratio (TBR) was measured for [^18^F]fluoromisonidazole (FMISO).

**Results:**

Median FDG TNR was 1.52 (interquartile range [IQR], 1.13–2.13) for GBM and 2.89 (IQR, 1.95–3.85) for PCNSL. MET TNR was 6.38 (IQR, 4.68–7.48) for GBM and 4.01 (IQR, 3.28–7.52) for PCNSL. FLT TNR was 17.35 (IQR, 11.10–22.28) for GBM and 25.61 (IQR, 15.91–55.30) for PCNSL. FMISO TBR was 2.72 (IQR, 2.22–3.62) for GBM and 1.58 (IQR, 1.04–1.93) for PCNSL. Receiver operating characteristic analysis showed areas under the curve of 0.78 (FDG), 0.62 (MET), 0.69 (FLT), and 0.85 (FMISO). FLT TNR had the highest sensitivity of 83.2% while FMISO TBR had the highest specificity of 84.2%. Among the four tracers, FLT showed the highest sensitivity and FMISO showed the highest specificity. Dual‑tracer combinations did not improve overall diagnostic accuracy beyond the best single tracers.

**Conclusions:**

Among four PET tracers, FMISO was most effective in distinguishing GBM from PCNSL. Diagnostic accuracy was highest when each tracer was used individually.

## Background

Gliomas are primary brain tumors that originate from glial cells, which provide structural and metabolic support to neurons in the central nervous system. Among gliomas, glioblastoma multiforme (GBM) is the most aggressive and malignant subtype based on histopathological classification. Primary brain tumors comprise a diverse group of neoplasms, with GBM and primary central nervous system lymphoma (PCNSL) being among the most common malignant types. GBM and PCNSL account for approximately 12 and 4% of all primary brain tumors, respectively. Despite their relative frequency, accurate diagnosis is essential, as these tumors exhibit distinct clinical behaviors and require different therapeutic strategies (Louis et al. 2021, 2016; Zhang et al. 2022).

Tumor diagnosis is typically confirmed by histopathological examination. In cases where biopsy is difficult, imaging and cerebrospinal fluid cytology can aid in the diagnosis of lymphoma (Glaudemans et al. 2013; Dondi et al. 2024). However, because GBM and PCNSL often exhibit similar imaging features, distinguishing between them can be challenging. Nonetheless, accurate differentiation is critical due to significant differences in treatment strategies and prognoses (Norikane et al. 2024; Flexner et al. 1994).

Positron emission tomography (PET) can be useful in differentiating PCNSL from GBM (Shinomiya et al. 2013; Cao et al. 2022; Lee and Scott 2007). Guidelines published by the International Primary CNS Lymphoma Collaborative Group have highlighted the value of [^18^F]fluoro-2-deoxy-D-glucose (FDG) PET, leading to its widespread use for detecting systemic neoplastic lesions (Hsu et al. 2022).

In addition to FDG, L[methyl[^11^C]]methionine (MET) and [^18^F]fluoromisonidazole (FMISO) may also aid in distinguishing PCNSL from GBM using PET (Lee and Scott 2007; Toh et al. 2013). However, comparative studies among these tracers remain limited, and their differential diagnostic performance is not well understood. Although 3′deoxy3′[^18^F]fluorothymidine (FLT) has been reported as useful for glioma grading, its effectiveness in differentiating GBM from PCNSL is unclear (He et al. 2021). Given prior reports of FLT accumulation in PCNSL, it is important to examine differences in FLT uptake between GBM and PCNSL (van der Meulen M et al. 2018). Limited availability of facilities capable of performing multi-tracer PET studies has restricted broader evaluation. Therefore, we aimed to assess the diagnostic utility of four PET tracers—FDG, MET, FLT, and FMISO—routinely used at our institution to differentiate PCNSL from GBM. In addition to previously reported combinations such as FDG and FMISO (Toh et al. 2013), we also investigated other tracer combinations.

## Methods

### Ethics

The Human Subjects Ethics Committee at Kagawa University Faculty of Medicine approved this retrospective, single-center study that included the use of FDG, MET, FLT, and FMISO PET tracers (Approval no: 2019–027). The study complied with the ethical principles enshrined in the Declaration of Helsinki (2013 amendment). This retrospective study was approved by the Human Subjects Ethics Committee at Kagawa University Faculty of Medicine (approval no. 2019‑027), and the requirement for written informed consent was waived because of the retrospective study design.

## Study design and population

**Fig. 1:**
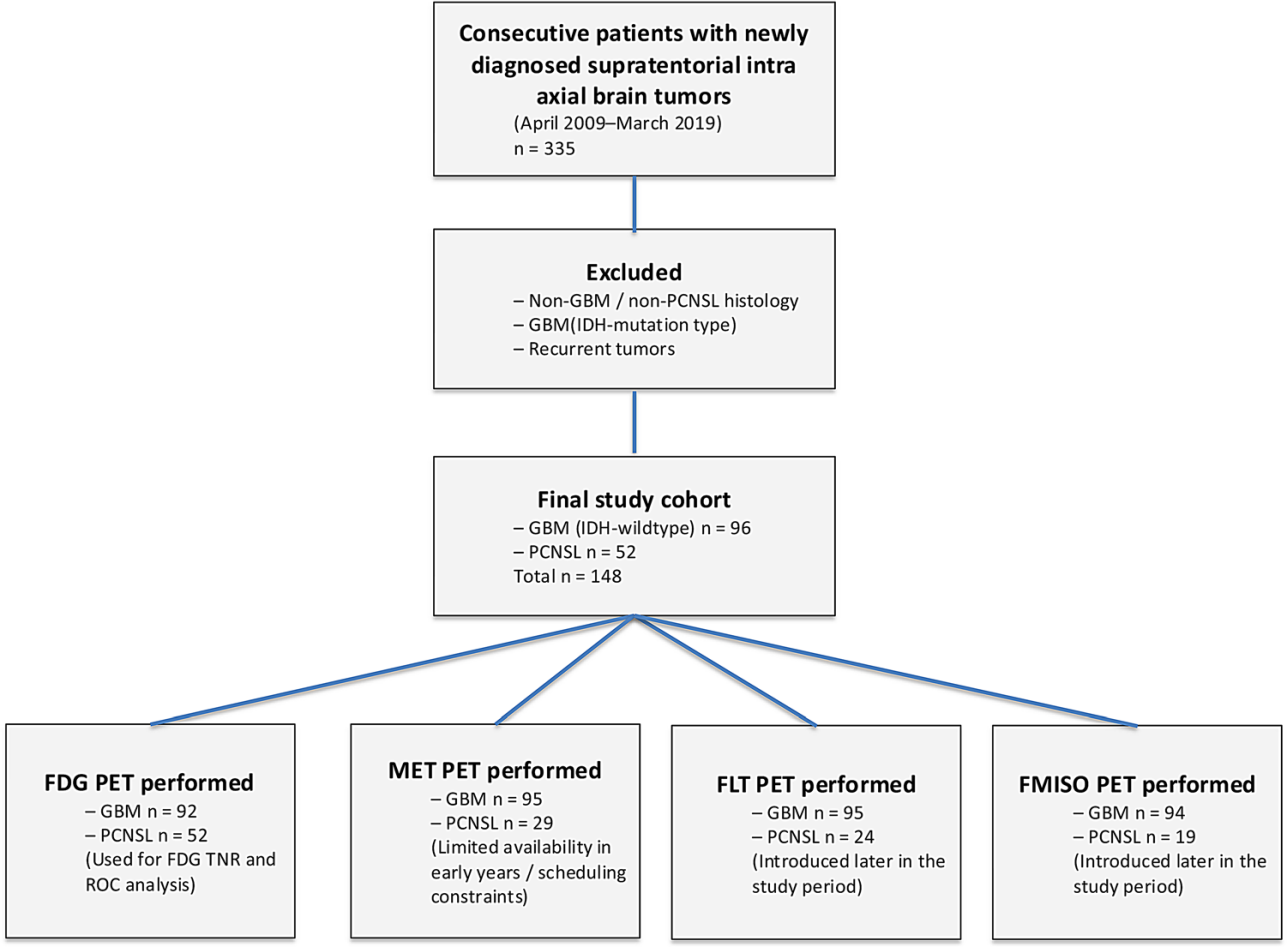
Flow diagram of patient selection and PET tracer allocation. Consecutive patients with newly diagnosed supratentorial intra-axial brain tumors (April 2009–March 2019) were screened (*n* = 335). After excluding non-GBM/non-PCNSL histologies, IDH-mutant GBM, and recurrent tumors, 148 patients were included in the final cohort (GBM IDH-wildtype, *n* = 96; PCNSL, *n* = 52). The diagram shows the numbers of GBM and PCNSL patients who underwent FDG, MET, FLT, and FMISO PET, respectively, reflecting tracer availability over time and clinical scheduling constraints. GBM, Glioblastoma; PCNSL, primary central nervous system lymphoma; FDG, [^18^F]fluoro-2-deoxy-D-glucose; MET, L[methyl[^11^C]]methionine; FLT, 3′deoxy3′[^18^F]fluorothymidine; FMISO, [^18^F]fluoromisonidazole

We retrospectively analyzed data acquired from patients with newly diagnosed supratentorial GBM or PCNSL between April 2009 and March 2019. The overall screening process, exclusions, and allocation of GBM and PCNSL patients to each PET tracer subgroup are summarized in Fig. 1. In this study, cases of IDH1-mutant type GBM were excluded from the analysis. All patients were preoperatively assessed using magnetic resonance imaging (MRI), including T2-weighted fluid-attenuated inversion recovery and gadolinium-enhanced T1-weighted sequences, followed by PET/computed tomography (CT) imaging with one or more tracers: FDG, MET, FLT, and FMISO. Pathological diagnoses were based on the 2016 World Health Organization classification (Louis et al. 2021). Lesions in the basal ganglia and brainstem were diagnosed based on quantitative FDG results or histopathological findings of biopsies, when available. In total, histopathological confirmation was not obtained in 9 of the 96 GBM cases and 23 of the 52 PCNSL cases. In the GBM group, the reasons for not performing a biopsy were anatomical inaccessibility (*n* = 4), high risk of hemorrhage (*n* = 3), poor general condition (*n* = 1), and other reasons (*n* = 1). In the PCNSL group, the reasons were anatomical inaccessibility (*n* = 14) and poor general condition (*n* = 9). In these non‑biopsied cases, the diagnosis was established based on characteristic MRI findings, and, for suspected PCNSL, was further supported by the kinetic parameter K3 obtained from dynamic FDG PET, which is routinely used at our institution as an ancillary criterion for confirming PCNSL. For patients with PCNSL, a review of available medical records confirmed that no corticosteroids were administered before the diagnostic PET examinations, in order to avoid steroid-related alterations in tracer uptake.

## MRI and PET/CT imaging

Patients underwent MRI using a MAGNETOM Skyra 3-T scanner (Siemens Healthcare, Erlangen, Germany). Gadolinium-enhanced axial T1-weighted images were acquired under the following parameters: repetition/echo time, 400/11 ms; slice thickness, 5 mm; matrix, 230 × 384.

We reconstructed 51 simultaneously acquired transverse 3D PET/CT images per field of view (FOV), at 3-mm intervals, in a total axial FOV of 15 cm using a Biograph mCT PET/CT scanner (Siemens Healthineers). The PET radiotracers were produced using an HM-18 cyclotron (Sumitomo Heavy Industries, Tokyo, Japan).

For FDG PET, patients were instructed to fast for at least 6 h before imaging, and serum glucose levels were analyzed before the injection of FDG, which was performed only if serum glucose was within normal levels. Each patient received an intravenous injection of FDG at 3.7 MBq/kg (mean, 235 ± 48.8 MBq). After 45–60 min, regional emission images of the brain were obtained for 10 min. For MET PET, a dose of 182–448 MBq (mean, 316 ± 55.6 MBq) of MET was injected intravenously at 6.0 MBq/kg and regional emission images were obtained for 5 min, beginning 10 min after MET administration. For FLT PET, the injected activity was adjusted on each scanning day because of the relatively short physical halflife of FLT and daytoday variability in production yield. In practice, the protocol was planned so that each patient received approximately 300–350 MBq of FLT. The actual injected activities in this cohort ranged from 141 to 398 MBq (mean, 302 ± 45.2 MBq), and regional emission images were obtained for 15 min, beginning 60 min after FLT administration For FMISO PET, no special dietary instructions were given to the patients. Regional emission scans were acquired for 10 min, beginning 120 min after an intravenous bolus injection of FMISO at 3.7 MBq/kg (mean, 262 ± 54.3 MBq). For FMISO PET, a venous blood sample was obtained during the emission scan. A 1‑mL whole‑blood sample was counted in a calibrated gamma well counter, and blood activity was expressed as MBq/mL and decay‑corrected to the time of injection. FMISO PET images were scaled to this venous blood concentration to produce voxel‑wise tumor‑to‑blood (T/B) values, allowing calculation of a tumor‑to‑blood ratio (TBR) map for each study. PET data were acquired in three-dimensional mode and reconstructed using an ordered-subsets expectation maximization (OSEM) algorithm with point-spread-function and time-of-flight modeling.

## PET/CT analysis

The uptake of FDG, MET, and FLT in brain tumors was semi quantified using standardized uptake values (SUVs). Regions of interest (ROI) were manually drawn around the portion of each lesion with the highest signal. The maximum SUV (SUVmax) was taken as a representative value for each tumor. The maximum tumor-to-normal tissue ratio (TNR) was determined by dividing the tumor SUVmax by the mean SUV of normal brain parenchyma, which was measured in contralateral normal-appearing cerebral white matter. ROI for the reference were carefully placed to avoid edema, suspected tumor infiltration, and sulcal cerebrospinal fluid.

For FMISO, tumor uptake was semi‑quantified using the TBR. A three‑dimensional region of interest was drawn over the enhancing tumor, and the maximum voxel value on the T/B map within this region was defined as the tumor TBR. The T/B map was obtained by dividing the PET activity concentration in each voxel by the decay‑corrected venous blood activity measured at the time of imaging.

The PET and MRI datasets were transferred to a Linux workstation, and MRI data were coregistered with FDG, MET, FLT, FMISO PET data using Dr. View/Linux, version R2.5 (AJS, Tokyo, Japan). All PET analyses were performed on a patient basis; in patients with multiple lesions, the lesion with the highest SUVmax was used for quantification.

## Statistical analysis

We compared tumor-to-normal tissue ratio (TNR) values for FDG, MET, and FLT, and tumor-to-blood ratio (TBR) values for FMISO between the GBM and PCNSL groups using the Mann–Whitney U test. The diagnostic utility of FDG, MET, and FLT TNRs and FMISO TBR in differentiating GBM from PCNSL was evaluated using receiver operating characteristic (ROC) curve analysis, with FDG TNR used as the reference. ROC curves for MET, FLT, and FMISO were compared to determine statistically significant differences. Cutoff values were defined as the point at which the sum of sensitivity and specificity (Youden index) was maximized. To further assess the combined diagnostic performance of the PET tracers, we constructed a multivariable logistic regression model with PCNSL as the dependent outcome (PCNSL = 1, GBM = 0). FDG TNR, MET TNR, FLT TNR, and FMISO TBR were entered simultaneously as continuous predictors. Odds ratios (ORs) and 95% confidence intervals (CIs) were obtained from this model to evaluate the independent association of each tracer with the diagnosis of PCNSL. Additionally, combinations involving FDG or MET—tracers commonly available at many institutions—were assessed for their diagnostic performance in combination with other tracers. For FDG and FLT, a positive result for PCNSL was defined as a tracer value above the cutoff derived from ROC analysis with PCNSL as the positive class. For MET and FMISO, ROC analysis was performed with GBM as the positive class, and therefore a positive result for PCNSL was defined as a tracer value below the corresponding cutoff. All statistical analyses were performed using JMP® Pro 17.0 (SAS Institute Inc., Cary, NC, USA), with *p*-values < 0.05 considered statistically significant.

At our institution, multi‑tracer PET is routinely used for the preoperative evaluation of brain tumors. In principle, all four tracers (FDG, MET, FLT, and FMISO) were planned for each newly diagnosed supratentorial lesion, and there was no institutional policy to preferentially select a specific tracer for particular clinical or radiological subtypes.

## Results

### Patient characteristics

A total of 148 patients were included, comprising 96 with GBM (median age: 70 years; range: 18–86 years) and 52 with PCNSL (median age: 70 years; range: 40–90 years). There were no significant differences in age or sex distribution between the two groups. The number of lesions observed on MRI was significantly higher in patients with PCNSL than in those with GBM (Table 1). All GBM cases were IDH1 wild-type, and all PCNSL cases were diagnosed as diffuse large B-cell lymphoma.

**Table 1:** Clinical characteristics of 148 patients

Clinical characteristics	GBM (n = 96)	PCNSL (n = 52)	p value
Age at diagnosis (y), median (min–max)	70 (18–86)	70 (40–90)	0.347
Sex, male/female	52/44	34/18	0.189
Lesions on MRI			
Single (%)	78.1%	59.6%	
Multiple (%)	21.9%	40.4%	
Number of lesions, mean±SD	1.5 ± 1.1	2.2 + 2.1	0.037

GBM, glioblastoma multiforme; PCNSL, primary central nervous system lymphoma; IQR, interquartile range; FDG, [^18^F]fluoro-2-deoxy-D-glucose; MET, L[methyl[^11^C]]methionine; FLT, 3′deoxy3′[^18^F]fluorothymidine; FMISO, [^18^F]fluoromisonidazole; MRI, magnetic resonance imaging; SD, standard deviation

## Analysis of PET/CT images

**Fig. 2:**
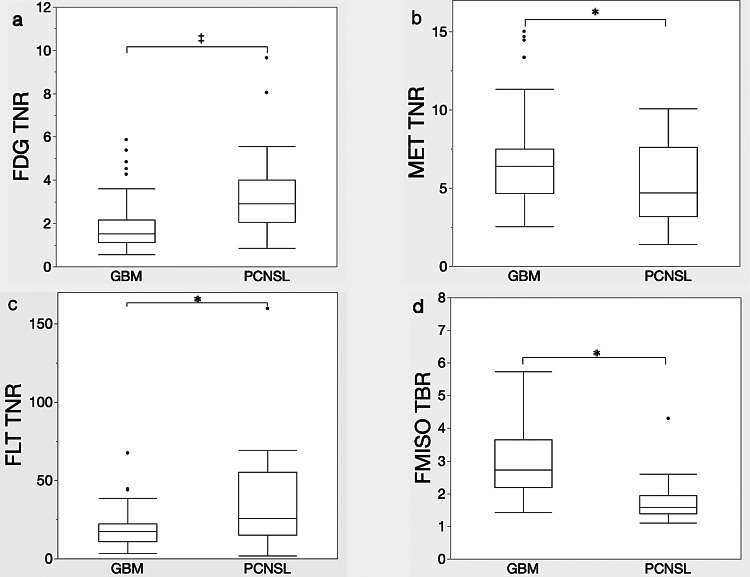
Box plots of three TNRs and of TBR using four tracers to differentiate GBM from PCNSL. (**a**–**c**): FDG, MET, FLT TNRs, respectively, and (**d**) FMISO TBR. Lines indicate averages and standard deviations; whiskers indicate minimum–maximum.*p < 0.05, ^†^*p* < 0.01, ^‡^*p* < 0.001. FDG, [^18^F]fluoro-2-deoxy-D-glucose; MET, L[methyl[^11^C]]methionine; FLT, 3′deoxy3′[^18^F]fluorothymidine; FMISO, [^18^F]fluoromisonidazole; PCNSL, primary central nervous system lymphoma; TBR; tumor-to-blood ratio; TNR, tumor-to-normal ratio; GBM, glioblastoma multiforme

All PET tracers showed significant differences in uptake between the GBM and PCNSL groups. The median FDG TNR was 1.52 (interquartile range [IQR], 1.13–2.13) for GBM (*n* = 92) and 2.89 (IQR, 1.95–3.85) for PCNSL (*n* = 52). The median MET TNR was 6.38 (IQR, 4.68–7.48) for GBM (*n* = 95) and 4.01 (IQR, 3.28–7.52) for PCNSL (*n* = 29). The median FLT TNR was 17.35 (IQR, 11.10–22.28) for GBM (*n* = 95) and 25.61 (IQR, 15.91–55.30) for PCNSL (*n* = 24). The median FMISO TBR was 2.72 (IQR, 2.22–3.62) for GBM (*n* = 94) and 1.58 (IQR, 1.04–1.93) for PCNSL (*n* = 19) (Table 2). FDG (*p* < 0.001) and FLT (*p* = 0.004) had significantly higher TNR values in PCNSL than in GBM, whereas MET (*p* = 0.046) and FMISO (*p* < 0.001) had significantly higher values in GBM than in PCNSL (Fig. 2).

**Table 2:** TNR for GBM and PCNSL

Characteristics	GBM		PCNSL	
	n	TNR median (IQR)	n	TNR median (IQR)
Tracer				
FDG	92	1.52 (1.13–2.13)	52	2.89 (1.95–3.85)
MET	95	6.38 (4.68–7.48)	29	4.01 (3.28–7.52)
FLT	95	17.35 (11.10–22.28)	24	25.61 (15.91–55.30)
FMISO	94	2.72 (2.22–3.62)*	19	1.58 (1.04–1.93)*

GBM, glioblastoma multiforme; PCNSL, primary central nervous system lymphoma; IQR, interquartile range; FDG, [^18^F]fluoro-2-deoxy-D-glucose; MET, L[methyl[^11^C]]methionine; FLT, 3′deoxy3′[^18^F]fluorothymidine; FMISO, [^18^F]fluoromisonidazole, *; Tumor to Blood ratio

## Diagnostic performance of PET tracers

**Fig. 3:**
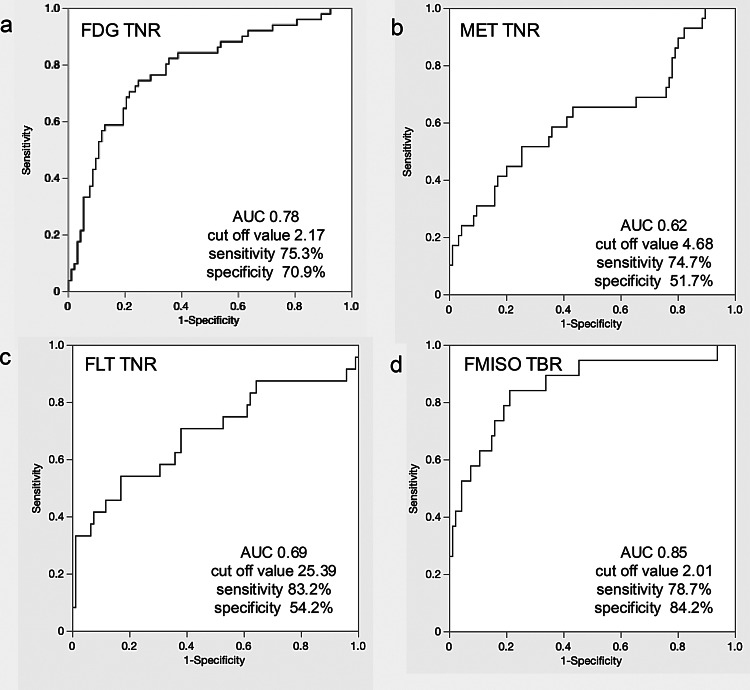
Receiver operating characteristic (ROC) curve analysis performed to differentiate GBM from PCNSL. (**A**) FDG TNR. (**B**) MET TNR. (**C**) FLT TNR. (**D**) FMISO TBR. FDG, [^18^F]fluoro-2-deoxy-D-glucose; MET, L[methyl[^11^C]]methionine; FLT, 3′deoxy3′[^18^F]fluorothymidine; FMISO, [^18^F]fluoromisonidazole; PCNSL, primary central nervous system lymphoma; TBR; tumor-to-blood ratio; TNR, tumor-to-normal ratio; GBM, glioblastoma multiforme

The areas under the ROC curves (AUCs) for FDG, MET, FLT TNR, and FMISO TBR were 0.785 (95% confidence interval [CI], 0.70–0.86; cutoff value, 2.17), 0.62 (CI, 0.49–0.74; cutoff, 4.68), 0.69 (CI, 0.54–0.83; cutoff, 25.39), and 0.85 (CI, 0.74–0.96; cutoff, 2.01), respectively. The sensitivity and specificity were as follows: FDG, 75.3% and 70.9%; MET, 74.7% and 51.7%; FLT, 83.2% and 54.2%; FMISO, 78.7% and 84.2% (Fig. 3). Among the four tracers, FLT had the highest sensitivity, while FMISO had the highest specificity.

**Fig. 4:**
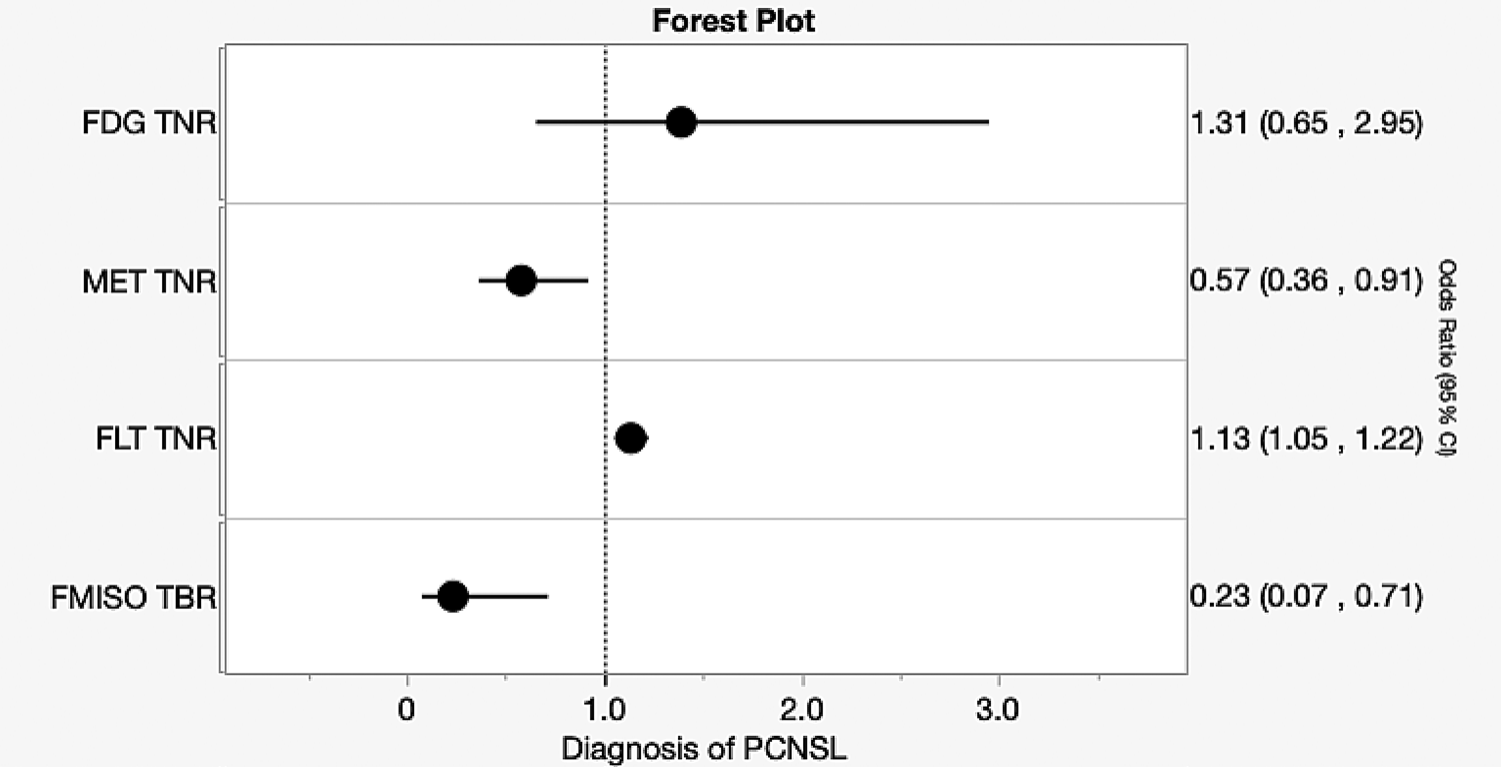
Forest plot of ORs and 95% CIs for each tracer in the diagnosis of PCNSL. CI, confidence interval; FDG, [^18^F]fluoro-2-deoxy-D-glucose; MET, L[methyl[^11^C]]methionine; FLT, 3′deoxy3′[^18^F]fluorothymidine; FMISO, [^18^F]fluoromisonidazole; ORs, odds ratios; PCNSL, primary central nervous system lymphoma; TBR; tumor-to-blood ratio; TNR, tumor-to-normal ratio; GBM, glioblastoma multiforme

The unit odds ratios (ORs) for the diagnosis of PCNSL were 1.31 (CI, 0.65–2.95) for FDG, 0.57 (CI, 0.36–0.91) for MET, 1.13 (CI, 1.05–1.22) for FLT, and 0.23 (CI, 0.07–0.71) for FMISO (Fig. 4). FDG did not reach statistical significance, while MET, FLT, and FMISO showed significant differences. In this multivariable model, the predicted probabilities yielded an AUC of 0.95, with a sensitivity of 0.94 and a specificity of 0.90; sensitivity and specificity were calculated at the cutoff that maximized the Youden index on the ROC curve.

Six combinations of dual tracers were evaluated (Table 3). Some combinations showed relatively high sensitivity or specificity, but none of the dual‑tracer combinations exceeded the AUC, sensitivity, or specificity of the best single tracers. Overall, diagnostic performance was highest when each tracer was used individually.

**Table 3:** Comparative diagnostic performance of ROC cutoff in PCNSL and GBM classification

（ROC cut off)	FDG(2.17) MET(4.68) GBM 96, PCNSL 28	FDG(2.17) FLT(25.39) GBM 92, PCNSL 24	FDG(2.17) FMISO(2.01) GBM 92, PCNSL 18
Sensitivity	0.565	0.685	0.620
Specificity	0.214	0.417	0.444

（ROC cut off)	MET(4.68) FLT(25.39) GBM 92, PCNSL 24	MET(4.68) FMISO(2.01) GBM 95, PCNSL 19	FMISO(2.01) FLT(25.39) GBM 93, PCNSL 18
Sensitivity	0.579	0.600	0.663
Specificity	0.250	0.069	0.500

GBM, Glioblastoma; PCNSL, primary central nervous system lymphoma; FDG, [^18^F]fluoro-2-deoxy-D-glucose; MET, L[methyl[^11^C]]methionine; FLT, 3′deoxy3′[^18^F]fluorothymidine; FMISO, [^18^F]fluoromisonidazole

## Illustrative cases

**Fig. 5:**
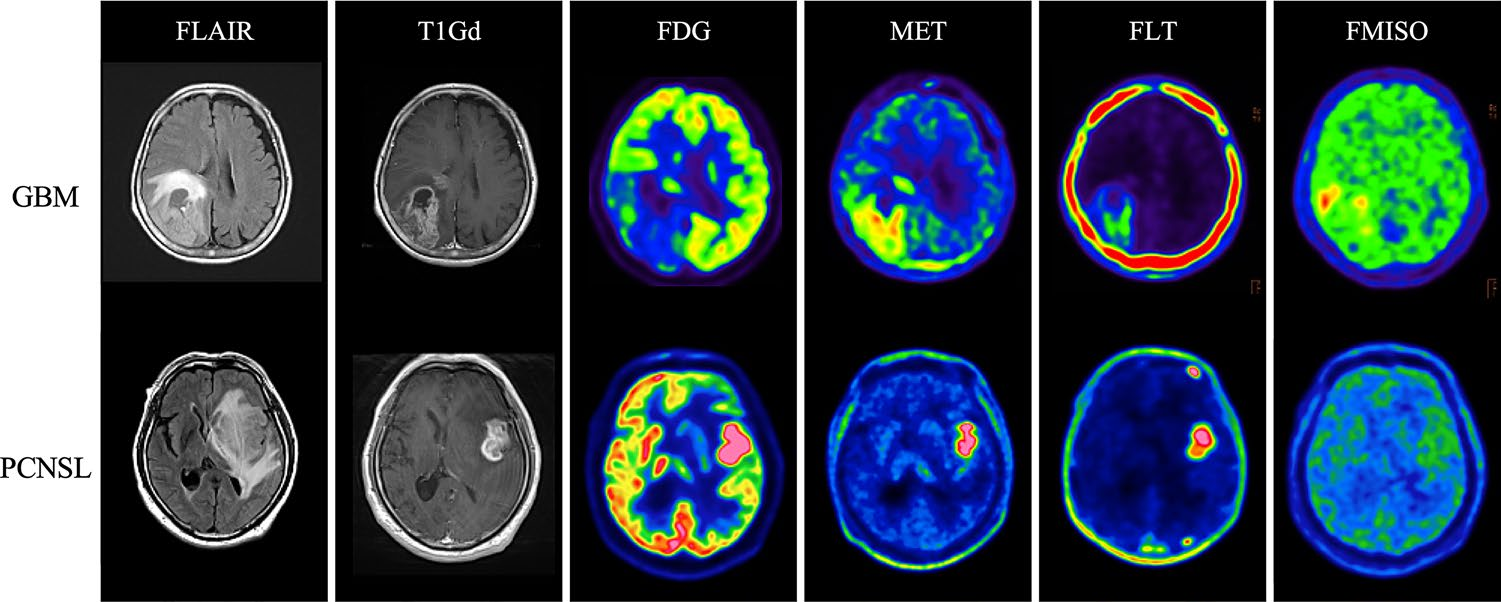
Representative cases of GBM and PCNSL with PET tracer uptake values. Upper panel: （Case1) a 71-year-old female with GBM. PET tracer uptake values (TNR and TBR): FDG 1.13, MET 4.60, FLT 8.43, FMISO (TBR) 3.05. Lower panel: (Case2) a 77-year-old male with PCNSL. PET tracer uptake values (TNR and TBR): FDG 2.59, MET 7.75, FLT14.68, FMISO (TBR) 1.45. FDG, [^18^F]fluoro-2-deoxy-D-glucose; MET, L[methyl[^11^C]]methionine; FLT, 3′deoxy3′[^18^F]fluorothymidine; FMISO, [^18^F]fluoromisonidazole; PCNSL, primary central nervous system lymphoma; TBR; tumor-to-blood ratio; TNR, tumor-to-normal ratio; GBM, glioblastoma multiforme

Case 1: A 71-year-old female with newly diagnosed GBM in the right parietal lobe. PET tracer uptake values (TNR or TBR) were as follows: FDG 1.13, MET 4.60, FLT 8.43, and FMISO 3.05. FMISO showed high uptake, indicating a strong likelihood of GBM. Surgical resection was performed, and the pathological diagnosis confirmed IDH1-wildtype GBM (Fig. 5).

Case 2: A 77-year-old male with newly diagnosed PCNSL in the left temporal lobe. PET tracer uptake values were FDG 2.59, MET 7.75, FLT 14.68, and FMISO 1.45. High uptake of FDG and FLT suggested PCNSL. A biopsy was performed, and the diagnosis was diffuse large B-cell lymphoma (Fig. 5).

## Discussion

This study evaluated the diagnostic performance of four PET tracers—FDG, MET, FLT, and FMISO—in differentiating between GBM and PCNSL. Significant differences in the uptake of the four tracers were observed between GBM and PCNSL. The uptake pattern of each tracer reflected the distinct characteristics of the two tumor types. Our findings provide valuable insights into the utility of these tracers for improving diagnostic accuracy in cases of brain tumors that are difficult to conclusively identify.

### Tracer performance and tumor characteristics

The significant differences observed in tracer uptake between GBM and PCNSL reflected the underlying biological characteristics of these tumors. Consistent with previous studies, FDG uptake was significantly higher in PCNSL than in GBM, which aligns with the typically higher metabolic activity of lymphomas compared to gliomas (Louis et al. 2016; Zhang et al. 2022). In contrast, elevated MET uptake in GBM likely reflects increased amino acid transport and protein synthesis associated with tumor proliferation. Tumor cells require the external supply of methionine, which is transported into cells by the sodium-independent L-transporter. This process is influenced by the intracellular metabolism of methionine and reflects proliferation activity (Glaudemans et al. 2013). In previous systematic reviews, no significant difference in MET TNR between GBM and PCNSL was reported (Dondi et al. 2024; Norikane et al. 2024). In the present study, as in previous reports, the MET TNR value was calculated from the SUVmax, and a significant difference was seen between GBM and PCNSL. The evidence for the usefulness of MET varies between studies, and the reason for the significant difference observed in the present study is not entirely clear (Kawase et al. 2011).

FLT uptake was significantly higher in PCNSL than in GBM. FLT is internalized by cells and phosphorylated by thymidine kinase 1, resulting in intracellular trapping (Flexner et al. 1994). The accumulation of FLT thus indicates thymidine kinase 1 activity, closely linked to cellular proliferation. PCNSL tends to show uniform FLT uptake due to its homogeneous cellularity, whereas GBM demonstrates variable uptake because of its heterogeneous histology, including necrotic and hypoxic regions (Shinomiya et al. 2013; Cao et al. 2022). As a result of these histological differences, PCNSL tends to show higher FLT uptake than GBM.

FMISO uptake was more prominent in GBM, indicating more extensive tumor hypoxia compared to PCNSL. FMISO accumulation reflects the extent of tissue hypoxia and associated metabolic changes (Lee and Scott 2007). GBM’s highly disorganized and inefficient vasculature contributes to severe and uneven hypoxia, while PCNSL, with less angiogenesis and more perivascular growth, shows relatively preserved oxygenation (Hsu et al. 2022). These vascular differences may explain the variation in FMISO uptake patterns. In contrast, PCNSL, primarily a B-cell-derived non-Hodgkin lymphoma, shows less pronounced vascular abnormalities (Toh et al. 2013; He et al. 2021). PCNSL tumor cells tend to infiltrate and proliferate around existing blood vessels, with less prominent angiogenesis and vascular disruption than GBM (Toh et al. 2013; He et al. 2021). Consequently, GBM tends to more commonly show widespread and severe hypoxia, while PCNSL may maintain a relatively uniform oxygen supply.

### Diagnostic accuracy and clinical implications

ROC curve analysis revealed differing diagnostic performances across tracers. FDG demonstrated favorable results (AUC 0.78; sensitivity 73%; specificity 70%), supporting its utility in distinguishing GBM from PCNSL (Zhang et al. 2022; van der Meulen M et al. 2018; Zhou et al. 2018; Zou et al. 2017). Although a statistically significant difference in MET TNR was observed (Norikane et al. 2024; Kawase et al. 2011), its diagnostic accuracy remained low (AUC 0.62), consistent with prior literature suggesting limited usefulness. Recent comparative work reported no significant difference in MET uptake between PCNSL and IDH‑wildtype GBM, with clearly inferior diagnostic performance compared with FDG (Norikane et al. 2024). Similarly, systematic reviews have suggested that MET PET may not be sufficient for reliably distinguishing between GBM and PCNSL (Dondi et al. 2024). No prior studies have documented the use of FLT for distinguishing GBM from PCNSL. In the present study, FLT exhibited the highest sensitivity (83.2%) among the four tracers evaluated. FLT offers superior clinical diagnostic utility due to its low uptake in surrounding brain tissue and high visual contrast, enabling clear tumor delineation (Chen et al. 0000). FMISO demonstrated the highest overall accuracy with an AUC of 0.85, followed by FDG (AUC, 0.78). Notably, FMISO exhibited a high specificity (84.2%) in distinguishing GBM from PCNSL.

The OR analysis further supported the diagnostic value of MET, FLT, and FMISO, with each showing significant differences between GBM and PCNSL. The discrepancy between significant differences in mean FDG TNR and non-significant ORs when comparing PCNSL and GBM may be attributed to the influence of outliers, particularly in the smaller PCNSL group. While comparisons of means are sensitive to extreme values, ORs are more robust to outliers. This inconsistency underscores the importance of employing multiple statistical approaches when analyzing PET imaging data for tumor differentiation, as relying on a single metric may not adequately represent the diagnostic value of an imaging modality.

It was found that diagnostic accuracy was higher when using a single PET tracer rather than combining two tracers. Previous studies have shown no significant difference between FDG alone and the combination of FDG and FMISO (Uchinomura et al. 2022) There is a possibility that changing cutoff values when combining tracers could increase diagnostic performance. Cutoff values defined for single-tracer diagnosis may not be appropriate.

FMISO showed the highest diagnostic accuracy, but clinical implementation is limited due to its availability. Development of hypoxia-targeting tracers with longer half-lives may help overcome this limitation and expand clinical accessibility.

Future research should include prospective, multi-center studies with larger cohorts. The integration of PET data with advanced MRI techniques and molecular profiling may further refine diagnostic models. Cost-effectiveness and feasibility studies are also essential to promote broader adoption of multi-tracer PET protocols.

At our institution, multi‑tracer PET is routinely used for the preoperative evaluation of brain tumors. In principle, all four tracers (FDG, MET, FLT, and FMISO) were planned for each newly diagnosed supratentorial lesion, and there was no institutional policy to preferentially select a specific tracer for particular clinical or radiological subtypes.

In practice, however, the number of tracers acquired per patient depended on clinical logistics. Patients with contrast‑enhancing lesions highly suggestive of GBM were usually scheduled for open craniotomy and tumor resection; in these cases, the surgical schedule often allowed sufficient time to complete the full PET protocol before surgery. In contrast, lesions suspected to be PCNSL on MRI, or lesions with low vascularity on cerebral angiography, were typically managed with stereotactic biopsy under local anesthesia. For many of these patients, histological confirmation was obtained quickly and systemic chemotherapy was initiated soon thereafter, limiting the opportunity to perform all planned PET tracers.

Furthermore, the tracers were introduced sequentially over the study period (FDG first, followed by MET, then FLT, and finally FMISO), which contributed to the smaller number of PCNSL patients examined with FMISO.

## Limitations

This study has several limitations. Its retrospective, single-center design may limit generalizability. Sample size variations for different tracers could introduce bias. Future multi-center studies using standardized imaging protocols and balanced cohorts are necessary to validate these findings.

A major limitation of our study is that the four tracers were not obtained in an identical set of patients. Although our institutional policy was to perform all four tracers in all cases whenever possible, clinical logistics and the need for rapid initiation of chemotherapy in PCNSL resulted in fewer tracers, especially FMISO, being completed in this group. As a result, the FMISO data in PCNSL are based on a relatively small and non‑random subset of patients, and the observed “hypoxia profile” may not fully represent the broader PCNSL population.

Therefore, while FMISO demonstrated high specificity and overall diagnostic performance in our cohort, these findings should be regarded as hypothesis‑generating rather than definitive evidence that FMISO is “the best” tracer among the four. Future prospective studies with standardized imaging protocols and more balanced tracer acquisition across GBM and PCNSL are needed to validate our results.

## Conclusions

Each PET tracer exhibited distinct accumulation characteristics reflective of underlying tumor biology. Among the four tracers, FMISO demonstrated the highest discriminatory power in differentiating GBM from PCNSL. Interestingly, the diagnostic accuracy was higher when using individual PET tracers compared to combinations, possibly due to overlapping or confounding signals when tracers are used together. These findings highlight the importance of selecting the most appropriate single tracer based on the clinical question and tumor characteristics.

## Data Availability

All datasets used during the current study are available upon reasonable request from the corresponding author.

## Abbreviations

AUC: Area under the receiver operating characteristic curve
FDG: [^18^F]fluoro-2-deoxy-D-glucose
FLT: 3′deoxy3′[^18^F]fluorothymidine
FMISO: [^18^F]fluoromisonidazole
GBM: Glioblastoma multiforme
HIF: Hypoxia-inducible factor
IDH: Isocitrate dehydrogenase
MET: L[methyl[^11^C]]methionine
MRI: Magnetic resonance imaging
PCNSL: Primary central nervous system lymphoma
PET: Positron emission tomography
ROC: Receiver operating characteristic
SUV: Standardized uptake value
TBR: Tumor-to-blood ratio
TNR: Tumor-to-normal ratio
